# Growth curve registration for evaluating salinity tolerance in barley

**DOI:** 10.1186/s13007-017-0165-7

**Published:** 2017-03-23

**Authors:** Rui Meng, Stephanie Saade, Sebastian Kurtek, Bettina Berger, Chris Brien, Klaus Pillen, Mark Tester, Ying Sun

**Affiliations:** 1 Computer, Electrical and Mathematical Science and Engineering Division, King Abdullah University of Science and Technology, Thuwal, 23955-6900 Saudi Arabia; 2 Biological and Environmental Science and Engineering Division, King Abdullah University of Science and Technology, Thuwal, 23955-6900 Saudi Arabia; 3 Australian Plant Phenomics Facility, The Plant Accelerator, University of Adelaide, Urrbrae, South Australia 5064 Australia; 4 Phenomics and Bioinformatics Research Centre, University of South Australia, Adelaide, South Australia 5001 Australia; 5 Department of Statistics, The Ohio State University, Columbus, OH USA; 6 Institute of Agricultural and Nutritional Sciences, Martin Luther University Halle-Wittenberg, Betty-Heimann-Str. 3, 06120 Halle, Germany

**Keywords:** Functional ANOVA model, High-throughput phenotyping, Nested association mapping, Plant growth, Spatial variation, Temporal variation

## Abstract

**Background:**

Smarthouses capable of non-destructive, high-throughput plant phenotyping collect large amounts of data that can be used to understand plant growth and productivity in extreme environments. The challenge is to apply the statistical tool that best analyzes the data to study plant traits, such as salinity tolerance, or plant-growth-related traits.

**Results:**

We derive family-wise salinity sensitivity (FSS) growth curves and use registration techniques to summarize growth patterns of HEB-25 barley families and the commercial variety, Navigator. We account for the spatial variation in smarthouse microclimates and in temporal variation across phenotyping runs using a functional ANOVA model to derive corrected FSS curves. From FSS, we derive corrected values for family-wise salinity tolerance, which are strongly negatively correlated with Na but not significantly with K, indicating that Na content is an important factor affecting salinity tolerance in these families, at least for plants of this age and grown in these conditions.

**Conclusions:**

Our family-wise methodology is suitable for analyzing the growth curves of a large number of plants from multiple families. The corrected curves accurately account for the spatial and temporal variations among plants that are inherent to high-throughput experiments.

**Electronic supplementary material:**

The online version of this article (doi:10.1186/s13007-017-0165-7) contains supplementary material, which is available to authorized users.

## Background

Analysis of salinity tolerance in plants is necessary for our understanding of plant growth and productivity under saline conditions. Generally, high salinity has a negative effect on plant growth, causing decreases in productivity. High levels of salts in the soil reduce the ability of plant root cells to absorb water, and high levels of salts inside a plant lead to toxicity. A comprehensive review on the physiological and molecular mechanisms of salinity tolerance at cellular, organ, and whole-plant levels is written by Munns and Tester [1]. To understand how plants cope with salinity, Rajendran et al. [2] quantified three mechanisms that wheat uses to increase its salinity tolerance: osmotic tolerance, ion exclusion, and tissue tolerance.

Nowadays, advanced technologies and equipment allow the collection of large and reliable datasets related to plant growth variables, such as daily shoot growth and elemental concentration. These datasets allow us to explore salt tolerance in plants with sophisticated statistical tools. Hunt [3] proposed plant growth analyses using exponential curves to describe the relative growth rate, which they derived from the absolute growth rate, correcting for initial plant sizes. The maximum potential relative growth rate was then applied to analyze the growth of a wide range of plant species [4]. Golzarian et al. [5] showed that shoot biomass can be accurately inferred from projected shoot area, which is the total sum of pixels collected via high-throughput imaging at The Plant Accelerator^®^. These techniques can be used to capture large amounts of data that can help explain how plants respond under abiotic stresses; for example, the effects of drought on barley introgression lines [6] and the effects of salinity on rice diversity panels [7]. In fact, Al-Tamimi et al. [7] fitted cubic smoothing splines to estimate the daily growth of rice plants under saline conditions grown at The Plant Accelerator^®^.

In this paper, we use a functional data analysis approach to study the effects of salinity on growth patterns of barley. The field of functional data analysis is a branch of statistics that is concerned with analyzing datasets involving continuous curves and surfaces. In this work, we restrict ourselves to statistical analysis of temporal growth curves of barley plants from a nested association mapping population that consists of 25 diverse inbred families called HEB-25 [8]. For further details about the HEB-25 population, refer to Maurer et al. [8]. An important challenge in this approach is to resolve the intra- and inter-family misalignment or misregistration of the important growth patterns (peaks and valleys) of the plants. There exists a large amount of literature on statistical analysis of 1D functions, namely the pioneering work of Ramsay and Silverman [9], Kneip and Gasser [10], and Tang and Müller [11]. Some specific applications include disease classification using cyclostationary biomedical signals [12], principal component analysis (PCA) for sparse longitudinal data [13], and classification of gene expression data [14]. When narrowing our focus to the analysis of functions that require temporal alignment, the literature is more limited [15–19]. The recent work of Srivastava et al. [20] and Kurtek et al. [21] provide a mathematically and statistically elegant approach for functional data registration (also referred to as amplitude-phase separation). The approach is based on the extension of the nonparametric Fisher–Rao metric and a convenient transformation called the square-root slope function. We use this method in conjunction with other functional data analysis tools to study family-wise salinity tolerance (FST) in the HEB-25 family.

**Fig. 1:**
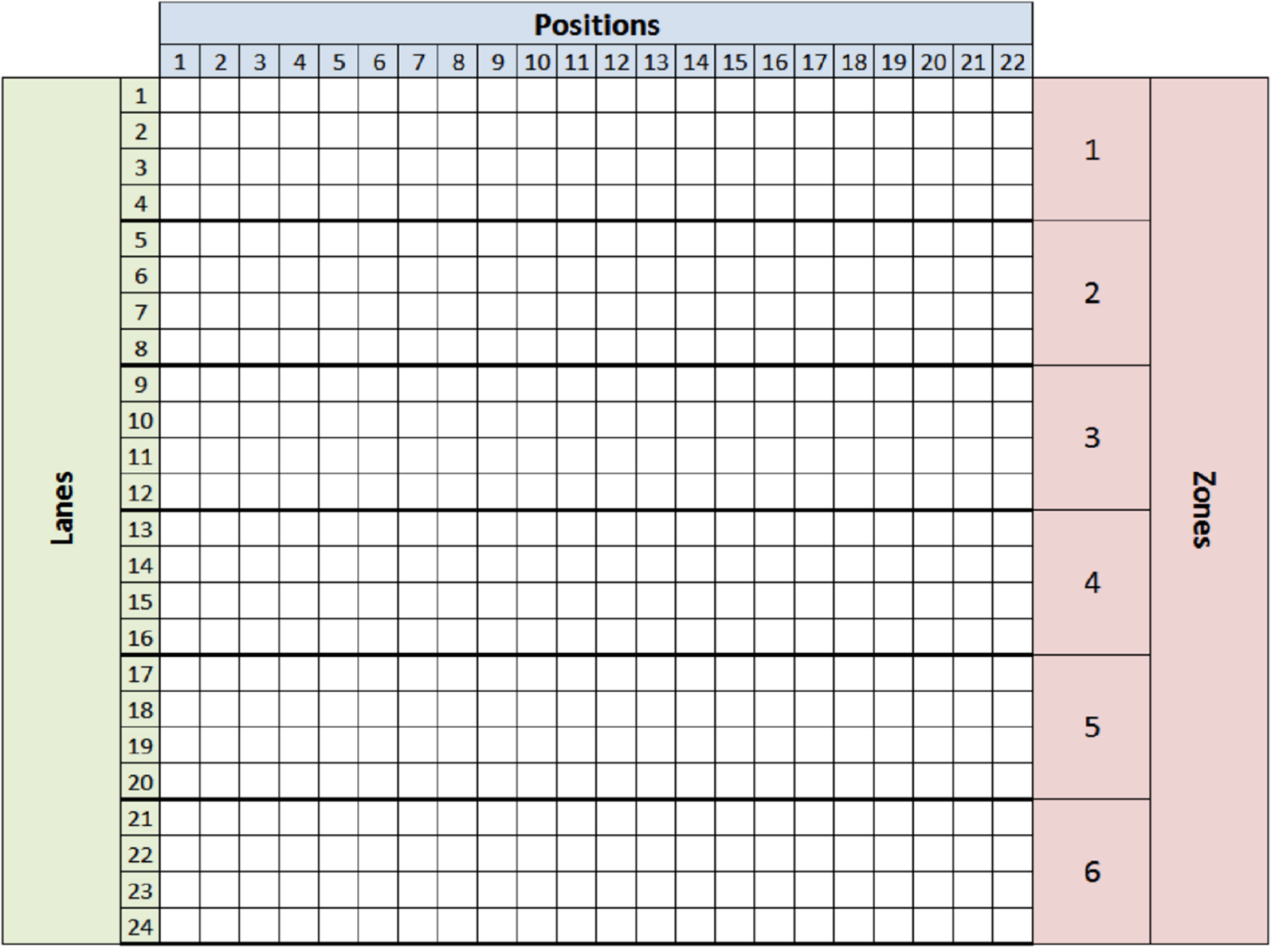
Design of the smarthouse. The smarthouse includes 24 lanes where each lane contains 22 positions, and four consecutive lanes are grouped as one zone

## Methods

The experiment was conducted in The Plant Accelerator^®^, a high-throughput phenotyping facility in Adelaide, Australia that includes northwestern (NW) and northeastern (NE) smarthouses. Each smarthouse has 24 lanes with 22 positions, and each four consecutive lanes are grouped as one zone due to homogeneous plant growth variability [22], dividing each smarthouse into a total of six zones. This setup is shown in Fig. 1. At each position, there is a cart that contains a pot with a single plant.

To minimize spatial variation, plant lines are allocated to main plots, which are pairs of positions with randomly assigned plants, and designated to be part of either the control (plants watered with rain water) or the treatment (plants watered with saline water) group. The lines are assigned to three runs throughout the year and two smarthouses. Table 1 summarizes the family allocation information and the number of lines for each family of the HEB-25 population. In addition, 36 main plots were allocated to Navigator, a local Australian line used as a check line. Because only the Navigator is replicated, spatial variations between and within smarthouses and temporal variation are estimated based on this check line.

**Table 1:** Summary of family allocation and number of lines per family, where 25 families (F01–F25) are randomly allocated to two smarthouses (NW and NE), and three runs

Smarthouse	NW			NE		
Run	1	2	3	1	2	3
Family	F09 (43)	F07 (55)	F10 (54)	F03 (66)	F02 (46)	F01 (52)
	F18 (22)	F11 (55)	F17 (54)	F04 (36)	F05 (54)	F06 (54)
	F20 (47)	F15 (55)	F24 (55)	F12 (65)	F16 (53)	F08 (51)
	F21 (46)	F19 (49)	F25 (54)	F13 (49)	F23 (52)	F14 (55)
	F22 (40)					

The number of lines per family is shown in the parentheses. There are two plants (control and saline) per line

First, four seeds per accession for each condition (control and saline) were sown, and watered to a gravimetric water content of 17%. At the two-leaf stage, the seedlings were thinned down to one plant per pot, while ensuring that the plant in the control pot is similar in growth and development to that in the saline treatment pot. Marble chips were added to the surface of the pot to reduce soil evaporation. The plants were loaded on to the conveyor belts in The Plant Accelerator^®^ at the time of emergence of the third leaf, about 16 days after sowing. After the appearance of the fourth leaf, about 20 days after sowing, we marked the third leaf and initiated the salt treatment by applying 200 mM NaCl to the treatment pots. After the stress imposition, daily images of the plants were taken for 14 days, using the LemnaTec Scanalyzer 3D, and the shoot biomass was inferred from the daily projected shoot area [5]. Fourteen days after salt imposition, the fully expanded fourth leaf was harvested and the sodium (Na) and potassium (K) contents per gram of leaf dry mass ($\upmu$mol/g DM) were measured by flame photometer to provide a measure of ion exclusion (Na) and retention (K). At the end of the experiment, a large dataset with 17 daily measurements, including Na and K contents of more than 3000 plants from 25 families and two experimental conditions were recorded. The phenotypic data is available as part of the Additional files 1, 2 and 3 for the three runs, respectively.

### Salinity sensitivity curves

In this section, we describe how to preprocess the data and define the salinity sensitivity (SS) curves. Let $x_{m\ell }(t)$ denote the number of pixels of the projected shoot area of the $\ell$th line in the *m*th family at time *t*, where $m = 1,\ldots ,26$, $\ell = 1$, $\ldots$, $n_m$, and $n_m$ is the total number of lines in the *m*th family. For *m*, 1 to 25 refer to the HEB-25 families and 26 refers to the Navigator. First, for each line, $x_{m\ell }(t)$ was smoothed by cubic splines [23] over the common time interval, $t\in [16,32]$ days. To account for the lines’ differing initial sizes, we scaled each growth curve by its initial size: $y_{m\ell }(t) = x_{m\ell }(t)/x_{m\ell }(16)$. Then, for each pair under control and saline conditions, we took the difference in plant size between the two conditions and divided it by the size of the line in the control condition, $z_{m\ell } = (y_{m\ell ,c} - y_{m\ell ,s})/y_{m\ell ,c}$. After smoothing the ratio by cubic splines, we predicted the first derivative denoted as $d_{m\ell } = z'_{m\ell }$. We then defined $d_{m\ell }$ to be the SS curve because it indicates how fast the relative difference, $z_{m\ell }$, changes over time. Oscillation values in $d_{m\ell }$ close to 0 suggest higher salinity tolerance, because this indicates that the growth of the plant under saline conditions was close to that under control conditions.

### Pairwise and multiple registration of salinity sensitivity curves

To align SS curves temporally, we used the general framework proposed by Srivastava et al. [20] and Kurtek et al. [21] due to its theoretical and practical advantages over other methods. We provide some details of this framework next. Let *f* denote an absolutely continuous, real-valued function defined on the temporal domain [16, 32] (i.e., a single observation of an SS curve). Let $\mathcal{F}$ denote the set of all such functions. Also, let $\Gamma =\{\gamma :[16,32]\rightarrow [16,32]|\gamma (16)=16,\ \gamma (32)=32,\ 0<\dot{\gamma }<\infty \}$ denote the set of temporal warping functions of the interval [16, 32], where $\dot{\gamma }=\frac{d\gamma }{dt}$. A temporal warping of an SS curve of *f* using $\gamma \in \Gamma$ is given by composition: $f\circ \gamma$. We seek a proper metric on $\mathcal{F}$ that provides tools for pairwise and multiple function alignment. The simplest idea is to use the standard $\mathbb {L}^2$ metric. In fact, this is the most common approach in the literature on function registration. Unfortunately, such an approach is not well suited to the function registration problem because $\Vert f_1-f_2\Vert \ne \Vert f_1\circ \gamma -f_2\circ \gamma \Vert$ for $f_1,\ f_2\in \mathcal{F}$ and $\gamma \in \Gamma$. In other words, the action of $\Gamma$ on $\mathcal{F}$ is not an isometry under the $\mathbb {L}^2$ metric. This theoretical deficiency has severe practical implications, including the pinching effect [24].

To overcome the previously described limitation, Srivastava et al. [20] used a different metric on $\mathcal{F}$ such that $d(f_1,f_2)=d(f_1\circ \gamma ,f_2\circ \gamma )$ which is known as the Fisher–Rao distance. This metric has many fundamental advantages, including the fact that it is invariant under temporal warping [25]; however, it is difficult to compute in practice. Therefore, we used a different representation of the original SS curves called the square-root slope function (SRSF), defined as $q = \text{ sign }(\dot{f})\sqrt{|\dot{f}|}$. It can be shown that if the SS curve of *f* is absolutely continuous, then the resulting SRSF is square-integrable (an element of $\mathbb {L}^2([16,32],\mathbb {R})$). Furthermore, if we temporally warp an SS curve *f* using a $\gamma \in \Gamma$, the SRSF of $f \circ \gamma$ is given by $(q,\gamma ) = (q \circ \gamma )\sqrt{\dot{\gamma }}$. The main motivation for using the SRSF representation for SS curves is that the complicated Fisher–Rao metric becomes the standard $\mathbb {L}^2$ metric and retains all of its desired properties, including isometry under the action of $\Gamma$. This result can be used to simply compute the Fisher–Rao distance $d_{FR}$ between any two SS curves as follows: $d_{FR}(f_1, f_2) = \Vert q_1 - q_2\Vert$, where $q_1$ and $q_2$ are the SRSFs of $f_1$ and $f_2$, respectively. Let $\mathcal{C} = \mathbb {L}^2([16,32],\mathbb {R})$ denote the space of all SRSFs. Then, for every $q \in \mathcal{C}$, there exists a unique SS curve of *f* such that $f(t) = f(16) + \int _{16}^t q(s) |q(s)| ds$. Thus, the representation $f \Leftrightarrow (f(16), q)$ is invertible. Note that because we use SRSFs (defined using the derivative of the SS curve), the temporal registration will be independent of the baseline (or vertical) variability of SS curves.

Our general approach to multiple registration of SS curves will be to jointly search for an average SS curve as well as the pairwise alignment of each SS curve in the sample to this mean. Thus, we begin by describing the pairwise registration approach. Define the equivalence class of an SRSF $q \in \mathcal{C}$ under the action of $\Gamma$ as $[q] = \{(q, \gamma ) | \gamma \in \Gamma \}$. Each equivalence class represents the set of SRSFs associated with all possible time warpings of a given SS curve. Similarly, any two SS curves in the set [*q*] differ only in their temporal alignment. Let $\mathcal{S}$ denote the set of all such equivalence classes (i.e., the quotient space $\mathcal{C}/\Gamma$). To compare any two equivalence classes, we will use the metric imposed on $\mathcal{C}$; given two SS curves $f_1$ and $f_2$, we register them using the $\mathbb {L}^2$ metric on the quotient space $\mathcal{S}$ using $d_\mathcal{S}([q_1],[q_2]) = \inf _{\gamma \in \Gamma } \Vert q_1 - (q_2 \circ \gamma )\sqrt{\dot{\gamma }} \Vert$. Note that this is a proper distance on this space (symmetric, positive-semidefinite, and satisfies triangle inequality). The minimizer of *d* is denoted by $\gamma ^*$ and represents the warping function that achieves optimal temporal alignment of $f_2$ to $f_1$. We also let $q_2^*$ denote $(q_2 \circ \gamma ^*)\sqrt{\dot{\gamma }^*}$ and $f_2^*$ denote $f_2\circ \gamma ^*$.

Next, we focus on mean estimation and multiple temporal alignment of SS curves. For a given collection of SS curves $f_1, f_2, \dots , f_n$, let $q_1, q_2, \dots , q_n$ denote their SRSFs, respectively. Then, the Karcher mean of the given SS curves is defined as $[\hat{\mu }] = \arg \min _{[q] \in \mathcal{S}} \sum _{i=1}^n d_\mathcal{S}([q],[q_i])^2$. We emphasize that the Karcher mean is actually an equivalence class $[\hat{\mu }]$ rather than an individual function. We choose a representative element of this equivalence class as follows. Select the element $\hat{\mu }\in [\hat{\mu }]$, which ensures that the mean of $\{\gamma _i^*\}$, the optimal warping functions aligning each SS curve in the given data to the Karcher mean, is the identity element of $\Gamma$ given by $\gamma _{id}(t)=t$. This is called the orbit-centering step. The full algorithm for computing the Karcher mean of functions is given in Srivastava et al. [20] and Kurterk et al. [21]. This procedure results in three items: (1) $\hat{\mu }$, the preferred element of the Karcher mean equivalence class $[\hat{\mu }]$; (2) $\{f_i^*\}$, the set of optimally registered SS curves; and (3) $\{\gamma _i^*\}$, the set of optimal temporal warping functions with mean $\gamma _{id}$.

**Fig. 2:**
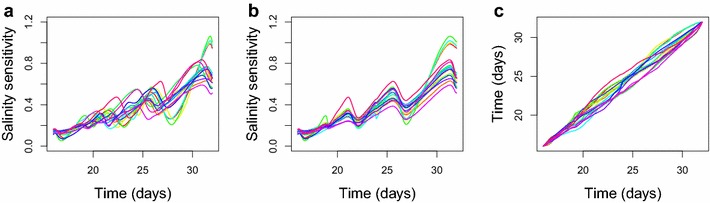
An example of curve registration. **a** The salinity sensitivity (SS) curves of the 16 functions from an arbitrary family, **b** SS curves after the curve registration, and **c** the corresponding time-warping functions. The salinity sensitivity on the y-axis of **a** and **b** refers to the derivative of the relative decrease in plant biomass

As a motivating example, we consider the 16 functions shown in Fig. 2. We suppose that these functions represent SS curves from one arbitrary family over the course of the experiment. Due to the natural variability in the response of plants to salinity stress, these functions clearly differ in relative heights and in the positions of their peaks and valleys. The time-warping method separates the amplitude and phase variabilities in Fig. 2 based on the Fisher-Rao Riemannian metric and using the square-root slope function representation to simplify the computation. The aligned functions display the relative heights of peaks and valleys, while the warping functions indicate their relative positions.

**Fig. 3:**
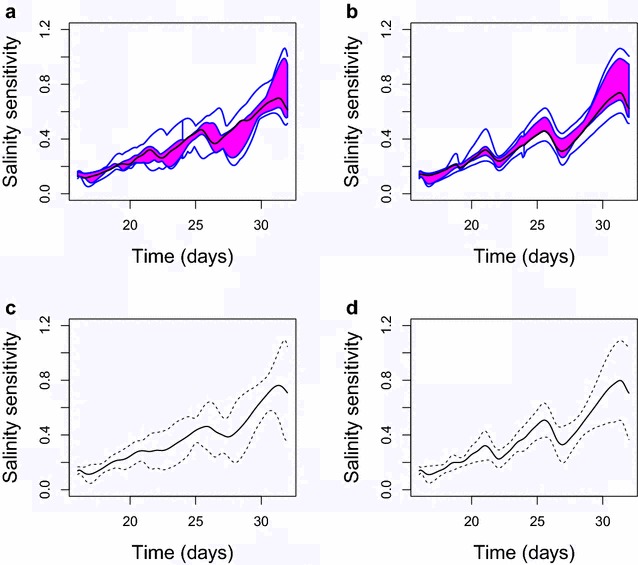
Summaries of the 16 salinity sensitivity (SS) curves before and after alignment. The plots show the functional boxplots of **a** the original curves and **b** the aligned curves, where the* solid black lines* in the middle represent the functional median. The point-wise means ± 2 standard deviations before and after the alignment are shown in **c** and **d**, respectively. The salinity sensitivity on the y-axis refers to the derivative of the relative decrease in plant biomass

Figure 3 shows the distributions of the original and the aligned functions. The point-wise means $\pm 2$ standard deviations are shown in the top panels, and the functional boxplots [26] are displayed in the bottom panels. In the functional boxplot, the black line is the functional median, which is the most representative function, and the box contains 50% of the most central functions. Both approaches demonstrate that the mean or the median of the aligned functions summarizes the patterns of the peaks and valleys with smaller variability than in the original functions.

In our analysis, we apply the time-warping technique to the available lines within each barley family and choose the aligned mean to represent the feature of a given family.

### Correcting location and time effects

Plant growth can be considerably affected by differences in microclimate conditions across and within smarthouses. For example, air temperature and humidity differ in different areas of a smarthouse depending on proximity to an air conditioning unit, causing the spatial variation described by Brien et al. [22]. Moreover, since the three runs happen during different times of the year, we propose a functional ANOVA model involving the variability in both locations (spatial) and runs (temporal) effects.

Let $d_{ijk\ell }$ be the SS curve of the Navigator from the *i*th run, *j*th room, *k*th zone, and $\ell$th plant, where $i=1,2,3$, $j=1,2$, $k=1,\ldots ,6$, and $\ell =1,\ldots ,6$. The model is

$$d_{ijk\ell } = \mu + \alpha _i + \beta _{jk} + \epsilon _{ijk\ell },$$

where $\mu$ represents the grand mean, $\alpha _i$ is the *i*th run effect, $\beta _{jk}$ is the location effect in the *k*th zone of the *j*th room, and $\epsilon _{ijk\ell }$ is an independent error process with mean 0. We estimate each item as follows:

1 $$\hat{\mu }= \frac{\sum _{i=1}^{3}\sum _{j=1}^{2}\sum _{k=1}^{6}\sum _{\ell =1}^{6}d_{ijk\ell }}{216},$$

2 $$\hat{\alpha }_{i}= \frac{\sum _{j=1}^{2}\sum _{k=1}^{6}\sum _{\ell =1}^{6}d_{ijk\ell }}{108} - \hat{\mu },$$

3 $$\hat{\beta }_{jk}= \frac{\sum _{i=1}^{3}\sum _{\ell =1}^{6}d_{ijk\ell }}{18} - \hat{\mu }.$$

**Fig. 4:**
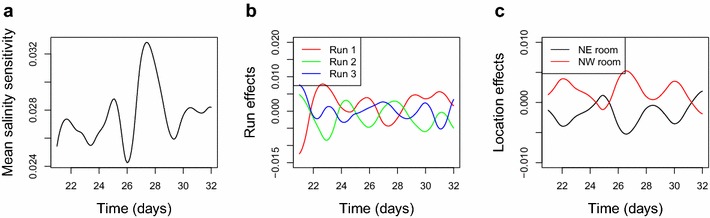
Estimated effects from the functional ANOVA model. We show **a** the grand effect, **b** the run effects and **c** the room effects. The salinity sensitivity on the y-axis of **a** refers to the derivative of the relative decrease in plant biomass

Then, Fig. 4 shows the estimated grand effect, run effects and room effects after adding the salt in the time interval $t\in [21,32]$, where the room effects are the averages of the zone effects within each smarthouse. Although we used the available data from Day 16 to Day 32 for growth curve analyses, only the time interval [21, 32] is considered for the salinity tolerance analysis, because we are interested in comparing the treated and untreated families only after the salt was added. The addition of salt was performed on Day 20, for which we do not have images, so the first day after salting is 21. In Fig. 4, the mean curve is always around 0.028, suggesting that plants are increasingly sensitive to salinity with increasing length of time. The effect curve of Run 1 is overall greater than the others, indicating that plants in Run 1 have relatively lower salinity tolerance. This might be because Run 1 was conducted during the summer when plants were exposed to the sun for longer than during other runs. The location effects show that the difference between the NE and the NW smarthouse is significant. Overall, plants in the NE smarthouse were less sensitive to salinity than plants in the NW smarthouse.

For convenience, we redefine $d_{mijk\ell }$ as the SS curve for the *m*th family, *i*th run, *j*th room, *k*th zone, and $\ell$th line. The corrected salinity sensitivity (CSS) curve is $c_{mijk\ell } = d_{mijk\ell } - \hat{\alpha }_i - \hat{\beta }_{jk}$.

### Family-wise salinity tolerance 

To summarize the salinity tolerance of different families, we applied, within each family, the multiple registration described in subsection “Pairwise and multiple registration of salinity sensitivity curves” to SS curves and CSS curves, and took the aligned mean to represent the growth pattern. To compare across families, we aligned the aligned means again to obtain the family-wise salinity sensitivity (FSS) curves and the corrected family-wise salinity sensitivity (CFSS) curves denoted by $f_m$ and $g_m$ and showing the change of the relative growth difference based on salinity condition.

**Fig. 5:**
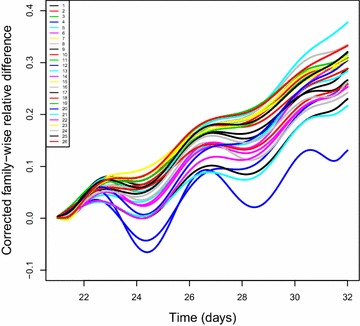
Corrected family-wise relative difference curves. Numbers 1,..., 25 refer to the HEB-25 families, and 26 refers to the Navigator. The corrected family-wise relative difference on the y-axis indicates the relative decrease in plant biomass corrected for each family

Taking the indefinite integral of $f_m$ and $g_m$ on time [21, 32] shows the growth relative difference directly. The resulting family-wise relative difference (FRD) curves and corrected family-wise relative difference (CFRD) curves are denoted with $F_{m}(t)$ and $G_{m}(t)$, $t \in [21,32]$. The calculation of the integral is essentially computing the area under the curve. A similar technique, called the “area under the disease progress curve” (AUDPC), was used in the study of plant disease resistance. Details can be found in Gilligan [27]. The CFRD curves are shown in Fig. 5, showing the relative difference at different times for the 25 HEB-families and for the Navigator. Therefore, we can compare the salinity tolerance for different families based on these corrected curves. For example, if the CFRD curves for family A are overall higher than the CFRD curves for family B, it implies that family A has a lower salinity tolerance than family B.

The traditional salinity tolerance index only considers the ratio of projected shoot area between saline and control conditions at the last day. We propose the family-wise salinity tolerance (FST) by integrating the corrected ratio $1-F_m(t)$ on [21, 32], and we propose the corrected family-wise salinity tolerance (CFST) by integrating the corrected ratio $1-G_m(t)$ on [21, 32]. Because a larger CFST suggests higher salinity tolerance, we evaluated the salinity tolerance of the 25-HEB families and the Navigator line by comparing their CFST values with their FST values.

## Element content analysis

**Fig. 6:**
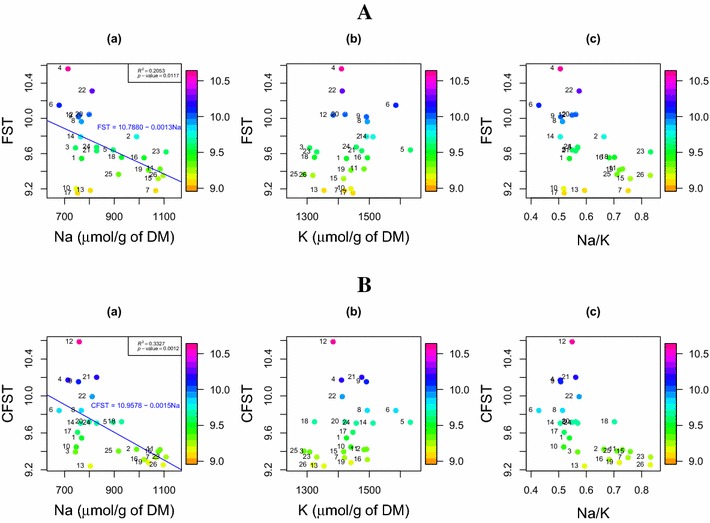
Plots of family-wise salinity tolerance versus element contents: **A** before correction (FST), and **B** after correction (CFST). The *y*-axis in **A**, FST, refers to family-wise salinity tolerance without corrections, and the *y*-axis in **B**, CFST, stands for the corrected family-wise salinity tolerance. The *x*-axis is *a* the Na content in $\upmu$mol/g of DM, *b* the K content in $\upmu$mol/g of DM, and *c* the Na/K ratio, respectively. Each *point* in these figures represents one family with the *color* indicating its salinity tolerance

This section discusses the relationship between sodium and potassium contents, and the FST before and after correcting the location and time effects. Figure 6 shows the relationship between CFST and FST of each family with the within-family averaged Na and K contents, as well as the Na/K ratios. The scatter plots are color-coded according to their salinity tolerance. As can be seen in Fig. 6B, the CFST is strongly negatively correlated with the contents of Na, while the relationship to K is not significant. A similar negatively related pattern is also observed for Na/K ratios, which suggests that Na contents dominate salinity tolerance in all families. After fitting a linear regression line, as shown in Fig. 6a, the linear relationship between CFST and Na is stronger ($R^2=0.33$) than that for the FST ($R^2=0.21$). In addition, we use the *t*-test to test how significant the slope is below zero. After correcting for location and time effects, the increase of $R^2$ indicates a much stronger negative linear relationship between salinity tolerance and Na contents. Therefore, it is necessary to remove or adjust for these types of environmental effects when evaluating the plant growth. Table 2 summarizes the $R^2$ and *p*-values when both linear and nonlinear regression models are fitted to each of the six cases in Fig. 6. For the nonlinear model, we fit a linear regression model to the logarithm of these salinity tolerance indices, which is equivalent to fitting exponential curves for these six cases. We can see that in all cases, the relationship between salinity tolerance indices and element contents becomes stronger after correction for both models we have considered, but only slightly so, and not in all cases. Therefore, we prefer to use simpler, linear relationships, especially as there is no a priori reason biologically, to expect these relationships to be exponential. In addition, there appears, by eye, to be a difference in the relationship between Na/K and CFST for Na/K values below 0.6, apparent in plot (c) of Fig. 6B. There also appears to be a similarly distinct relationship between Na and CFST, as seen in plot (a)–differing at about 850 $\upmu$mol/g DM. There may be a biological reason for this, where shoot Na is related to salinity tolerance at high values of Na, but not at low values of Na. Although this can make intuitive sense, at this stage we cannot take this further than noting it as a possible phenomenon.

**Table 2:** Summary of the $R^2$ and *p-*values when both linear and nonlinear regression models are fitted to each of the six cases in Fig. 6

	Panel plots	Before correction			After correction		
		A(a)	A(b)	A(c)	B(a)	B(b)	B(c)
		FST-Na	FST-K	FST-Na/K	CFST-Na	CFST-K	CFST-Na/K
Linear regression	$R^2$	0.2371	0.0573	0.2525	0.3594	0.0587	0.3688
	*p-*value	0.0117	0.2385	0.0089	0.0012	0.2332	0.0010
Log scale (exponential)	$R^2$	0.2337	0.0593	0.2505	0.3665	0.0627	0.3781
	*p-*value	0.0124	0.2305	0.0092	0.0011	0.2172	0.0008

Log scale (exponential) indicates that linear regression models are fitted to the logarithm of the salinity tolerance indices

## Discussion

In this paper, we applied a set of advanced statistical tools for analysis of the barley growth curves in response to salinity. We used relative difference in growth rate between plants under control and saline conditions as an indicator of salinity tolerance. In addition, the FST values were corrected to account for spatial variation among plants in a smarthouse and for the temporal variation associated with high-throughput experiments. The growth pattern is summarized for the HEB-25 families and the Navigator line. Because different lines within the same family often do not respond to salinity at the same time, curve registration techniques were applied through time-warping, such that averaging aligned lines better display family-wise features. This method is suitable for analyzing growth curves of a large number of plants from multiple families, while accounting for the spatial and temporal variations inherent to high-throughput experiments. It can also be used for experiments with similar designs but other stressors. In addition, our proposed CFST value allows a better understanding of the relationship between salinity tolerance and plant traits, such as the relationship between plant growth and Na and K contents, and the Na/K ratio. Although we proposed the CFST in our analysis, the curve registration technique can be used for any other functional indices of salinity tolerance as well if misalignment is an issue.

## Additional files

**Additional file 1.** Dataset for Run 1.

**Additional file 2.** Dataset for Run 2.

**Additional file 3.** Dataset for Run 3.
